# Biomarkers of obesity-mediated insulin resistance: focus on microRNAs

**DOI:** 10.1186/s13098-023-01137-3

**Published:** 2023-08-04

**Authors:** Yichen Cai, Pan Liu, Yumei Xu, Yuguo Xia, Xiaowan Peng, Haiyan Zhao, Qiu Chen

**Affiliations:** 1 Hospital of Chengdu University of Traditional Chinese Medicine, Chengdu, 610072 Sichuan China; 2 School of Clinical Medicine, Chengdu University of Traditional Chinese Medicine, Chengdu, China

**Keywords:** Biomarker, Insulin resistance, MicroRNAs, Obesity

## Abstract

Obesity and metabolic syndromes are becoming increasingly prevalent worldwide. Insulin resistance (IR) is a common complication of obesity. However, IR occurrence varies across individuals with obesity and may involve epigenetic factors. To rationalize the allocation of healthcare resources, biomarkers for the early risk stratification of individuals with obesity should be identified. MicroRNAs (miRNAs) are closely associated with metabolic diseases and involved in epigenetic regulation. In this review, we have summarized the changes in miRNA expression in the peripheral circulation and tissues of patients and animals with obesity-associated IR over the last 5 years and identified several candidate biomarkers that predict obesity-related IR. There are areas for improvement in existing studies. First, more than the predictive validity of a single biomarker is required, and a biomarker panel needs to be formed. Second, miRNAs are often studied in isolation and do not form a network of signaling pathways. We believe that early biomarkers can help clinicians accurately predict individuals prone to obesity-related IR at an early stage. Epigenetic regulation may be one of the underlying causes of different clinical outcomes in individuals with obesity. Future studies should focus on objectively reflecting the differences in miRNA profile expression in individuals with obesity-related IR, which may help identify more reliable biomarkers. Understanding the metabolic pathways of these miRNAs can help design new metabolic risk prevention and management strategies, and support the development of drugs to treat obesity and metabolic disorders.

## Challenges in treating obesity and its complications

**Fig. 1:**
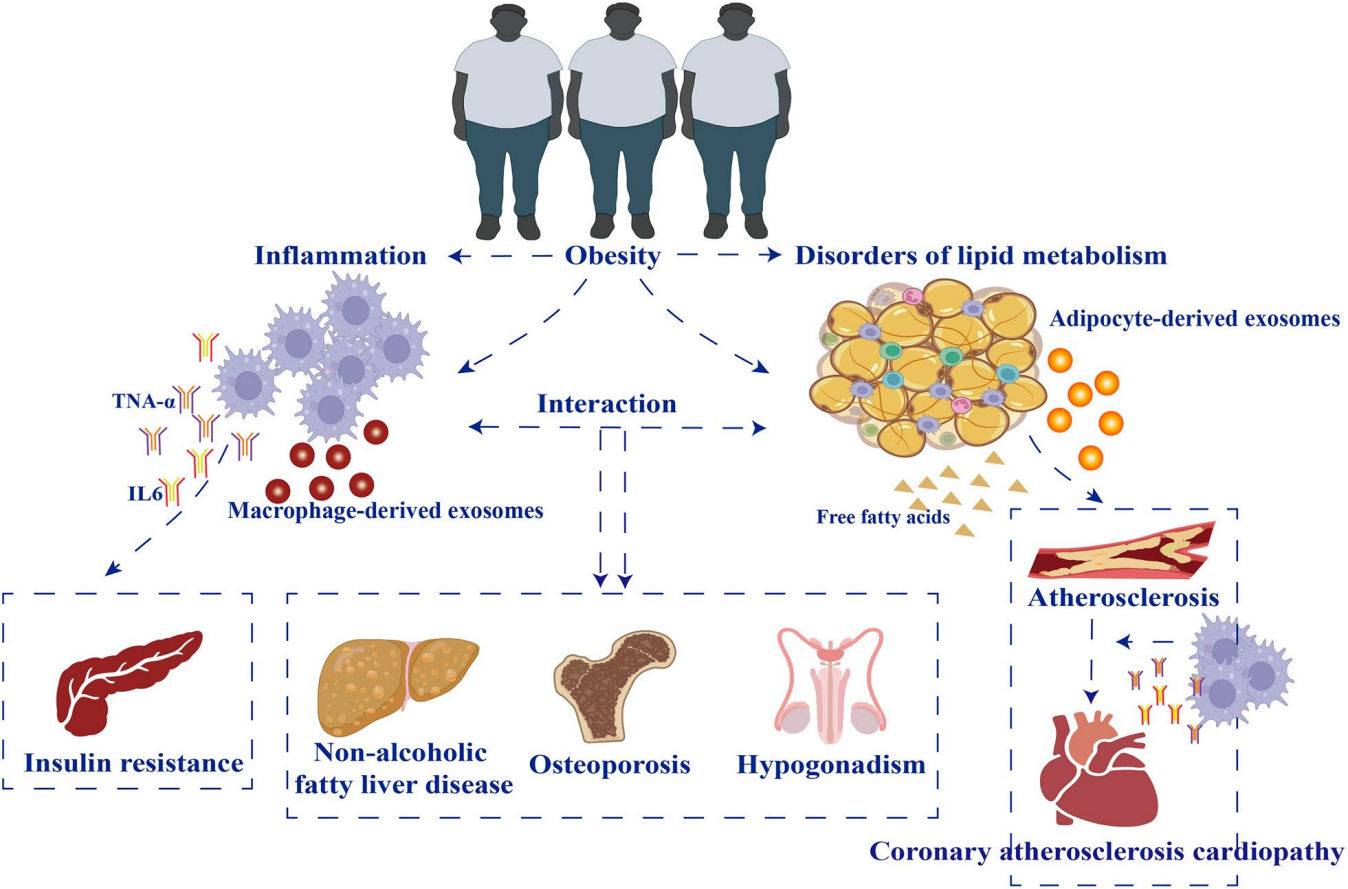
Complications caused by obesity. Obesity is a risk factor for many diseases, such as atherosclerosis, cardiovascular disease, insulin resistance, type 2 diabetes, hypogonadism, chronic inflammation, non-alcoholic fatty liver disease, osteoporosis, etc. There is an interrelationship between these complications. Obesity leads to chronic inflammation and disorders of lipid metabolism, where chronic inflammation is an intermediate factor leading to insulin resistance. Lipid metabolism disorder is an intermediate factor leading to atherosclerosis, while chronic inflammation will further lead to the development of atherosclerosis to coronary heart disease. In addition to this, chronic inflammation interacts with lipid metabolism disorders and both together lead to osteoporosis, hypogonadism and non-alcoholic fatty liver disease

Obesity prevalence has increased worldwide and reached epidemic proportions over the past few decades [1, 2]. Due to the complexity of the pathophysiological aspects of the disease, obesity cannot only be considered an energy imbalance between caloric intake and expenditure [3]. In addition to the dangers caused by obesity itself, it induces many complications, such as insulin resistance (IR), chronic inflammation, and atherosclerosis, which increase the risk of several diseases, such as metabolic syndrome, type 2 diabetes (T2D), and cardiovascular disease [4]. Approximately 4 million people die each year from obesity-related complications [4] (Fig. 1). Although clinicians have attempted to prevent obesity progression, its management remains challenging.

Among obesity-induced metabolic syndromes, we have focused on IR. Obesity and IR are closely interrelated and IR is a liaison between obesity and other obesity-related metabolic diseases [5]. Obesity mediates the development of other obesity-related complications, such as T2D [6], hypertension [7], obstructive sleep apnea syndrome [8], and male hypogonadism [9], by decreasing sensitivity to insulin and causing IR. Obesity-mediated insulin metabolism regulation involves several complex pathways, such as inflammatory pathways [10], mitochondrial dysfunction [11], gut microbial dysregulation [12], and fatty extracellular matrix remodeling [13]. However, a unified conclusion has yet to be reached. By summarizing the existing mechanisms, the endocrine and paracrine effects of adipocytes clearly play an essential role in IR development in individuals with obesity [14]. Adipose tissue is a storage site for extra energy molecules and is a metabolic organ. In the obese state, dysfunctional adipocytes produce large amounts of adipokines through endocrine function, which affect the function of tissues and organs throughout the body, and these adipokines are closely related to obesity-related metabolic diseases [15]. For example, Moreno-Navarrete et al. [16]. Showed that lipopolysaccharide-binding protein (LBP) is a novel adipokine. In the obese state, the synthesis and release of LBP increased with adipocyte differentiation in human and mouse adipose tissue. And there is a strong correlation between circulating concentrations of LBP and obesity-related metabolic disorders (especially IR) [17]. Rodríguez et al. [18] found increased adipocyte apoptosis in human obesity and obesity-associated T2D. Adipocyte apoptosis contributes to macrophage infiltration into adipose tissue, IR, and hepatic steatosis associated with human obesity [19]. In the obese state, ghrelin and ghrelin* O*-acyltransferase are co-produced in human visceral adipocytes and stromovascular fraction cells [18]. Acylated and desacyl ghrelin directly act on visceral adipose tissue to regulate TNF-α-induced apoptosis and autophagy in humans [18]. In summary, understanding the potential role of multiple adipokines in the obese state can help uncover important targets for obesity-related metabolic complications. miRNAs control critical steps in adipocyte differentiation, proliferation and browning, as well as lipolysis, lipogenesis and adipokine secretion [15]. miRNAs are mainly found in exosomes. Dysfunctional adipocytes release exosomes that transport bioactive substances (including proteins, lipids, and nucleic acids) to the surrounding target organs and peripheral plasma via paracrine effects. These exosomes mediate cell-to-tissue crosstalk [20–23]. In the obese state, exosomes secreted by adipocytes leak into the peripheral circulation, and some chemokines contained in exosomes may induce the aggregation of immune cells such as monocytes, thus causing inflammation [21]. For example, Catalán et al. [24] showed that the inflammatory protein calprotectin is elevated in the obese state and acts as a chemokine to induce macrophages to accumulate in adipose tissue, thus mediating a role in obesity-associated T2D. Under the paracrine action of adipocytes, pro-inflammatory macrophages accumulate in adipose tissue [25, 26] and exosomes secreted by macrophages leads to IR in adipocytes, a finding that has been demonstrated in an in vitro model [22]. Exosomes secreted by adipocytes and exosomes secreted by macrophages in individuals with obesity are jointly involved in the development of chronic inflammation and IR associated with obesity [27]. These exosomes contain a large number of miRNAs, which differ in each of these exosomes under the regulation of different epigenetic mechanisms, and this is one of the important reasons for the different complications in two patients with the same degree of obesity [28]. At the same time, this may explain the differences in the degree of IR among individuals with obesity.

From another perspective, we noted a high degree of variability among individuals with obesity. The individuals with obesity and metabolic syndrome have been referred to as metabolically abnormal obese (MAO) and those who do not develop metabolic syndrome are referred to as metabolically healthy obese (MHO). The cause for this distinction may be explained by epigenetics [29]. Epigenetics refers to changes in phenotype that are not rooted in DNA sequence [30]. The epigenome includes DNA methylation, histone modification, and non-coding RNAs (including a variety of RNAs with known functions such as long-stranded non-coding RNAs and miRNAs, as well as RNAs with unknown functions) [29]. They play a role in regulating cell differentiation and promoting cell-specific gene expression. The metabolic state and nutritional requirements of the body may be influenced by epigenetic processes, such as chromatin arrangement, which can stimulate structural adaptations that control gene expression. Therefore, epigenetic modifications are key mechanisms linking obesity and metabolic diseases [17]. The development of disease-associated gene- and genome-based new tools is essential for the early identification of the characteristics of individuals with obesity, which will help to screen individuals with a high prevalence of metabolic diseases at an early stage and enable targeted treatment for this population.

In addition, from a clinical and epidemiological point of view, individuals with obesity at a higher risk of developing metabolic complications should be identified so that the limited resources can be used more effectively for intensive treatment. Therefore, practical early screening tools are critical. Clinical tools for early disease screening include imaging and biomarkers (blood tests, urine tests, etc.). More elaborate techniques such as magnetic resonance imaging are increasingly available to assess body fat distribution, but these measures are not readily available in routine clinical practice, and health-relevant cut-offs not yet been established. The measurement of biomarkers that reflect the underlying biological mechanisms for the increased disease risk may be an alternative approach to characterize the relevant obesity phenotype. Therefore we focus on biomarkers. Biomarkers are biochemical indicators that mark changes or possible changes in the structure or function of systems, organs, tissues, cells, and sub-cells, and have various uses including aiding diagnosis [31], identifying disease subtypes [32], predicting disease prognosis [33], and assisting drug development [34]. Sensitive biomarkers can help clinicians make accurate judgments and predictions early in the disease process [35]. There are numerous types of biomarkers available, including but not limited to proteins [36], exosomes [37], and miRNAs [38], among which miRNAs are intimately involved in epigenetic regulatory processes. Since obesity-related IR is closely related to epigenetic regulation, we focused on miRNAs among many biomarkers. MiRNAs are a class of small post-transcriptional non-coding RNAs that regulate gene expression. Recently, miRNAs have received much attention as not only biological process regulators, but also predictive biomarkers in obesity management [39]. Mining MAO-specific miRNAs could facilitate early individualized prediction, prevention, and control of the epidemic of obesity-related complications.

In this review, we first introduce the general characteristics of miRNAs and the advantages and feasibility of miRNAs as biomarkers. We then describe miRNAs that have the potential to serve as biomarkers of obesity-related IR and summarize the current evidence from clinical studies and animal experiments regarding the association of these miRNAs with the risk of obesity-related IR.

## General aspects of miRNAs

miRNAs are epigenetically regulated 19–25 nucleotide long non-coding single-stranded RNAs. miRNAs promote cell-specific gene expression, while keeping the DNA sequence intact. This may be one of the underlying reasons for the high individual variation in individuals with obesity [40]. miRNAs are involved in the pathogenesis of many diseases, including cancer and metabolic disorders [38, 41]. Thus, although the miRNA functions are not fully defined yet, their large number and wide species distribution suggest that they play a crucial role in gene regulation [42]. Focusing on miRNA changes can help understand the causes of inter-individual differences in individuals with obesity and make targeted prevention efforts at an early stage.

The human endocrine system is homeostatic and the metabolic balance is disrupted in heterogeneous diseases such as obesity. In the early stages of a complex illness, biomarkers can reflect the complex disease process pathways and dimensions to derive alternative endpoints and provide a comprehensive assessment of treatment efficacy [43]. miRNAs have gradually gained widespread attention as biomarkers and may be involved in intercellular communication alone or as significant components of exosomes. They can function in the intracellularly cytoplasmic compartment and are stable in the extracellular environment [44]. miRNAs are widely distributed in living organisms and have been detected in many biological fluids, such as plasma, and urine [45]. In addition, miRNAs can significantly change in various organs, such as the liver, skeletal muscle, and adipose tissue [45]. These properties of miRNAs enhance the possibility of miRNAs being commonly detected as biomarkers in clinical settings. At present, miRNAs from blood or derived fractions are particularly interesting candidates for routine laboratory applications, as they can be measured in most clinical laboratories already today. This assures a good accessibility of respective tests. However, the choice of different assays may affect the results of miRNA detection [46]. To date, several methods have been developed to detect miRNAs. But most (pre)clinical studies dealing with miRNA expression profiling are performed with either of these three technologies: next generation sequencing (NGS) [47], microarrays [48], or reverse transcription polymerase chain reactions (RT-qPCR) [49], respectively. NGS can quantify known miRNA sequences, while it can identify and quantify previously unknown sequences [46]. NGS is therefore a valuable approach for both the discovery and the subsequent validation of novel diagnostic or prognostic miRNA signature. Moreover, NGS allows the multiplexed expression analysis of miRNA from different samples in a single experiment, thus eliminating factors that may negatively impact test results. Another clear advantage of NGS is its high dynamic range, which allows to accurately quantifying both highly expressed and low abundant miRNAs at the same time in the same experiment. Despite its many advantages, NGS data evaluation has not been standardized and has not yet become a routine method in the laboratory [50]. Microarrays are often used for miRNA screening assays, leading for example to the identification of disease miRNA signatures [51]. Despite being high-throughput and highly multiplexed, classic miRNAs microarrays display a relatively low sensitivity, with detection limits within the nanomolar range [48]. Notably, microarray technology cannot distinguish between mature miRNAs and their precursors, which may affect the results of expression analysis studies [52]. In addition, the extremely high similarity of miRNA sequences can lead to errors in microarray detection results, which may result in inconsistent results between microarray experiments and other miRNA expression profiling platforms [53, 54]. RT-qPCR is the gold standard for gene expression quantification [49]. In order to quantify miRNA expression by RT-qPCR, the cycling threshold of each miRNA measured must be correlated with the standard, suggesting that different endogenous controls during the experiment may lead to different diagnostic results, thus causing quantitative bias [55]. Collectively, for the discovery and validation of new miRNA signatures, NGS or microarray technology is more suitable. However, in a systematic comparison of different commercial platforms used for miRNA expression profiling, including microarrays, NGS and RT-qPCR. RT-qPCR tests exhibit the highest sensitivity and the best balance of sensitivity, specificity, accuracy and reproducibility [56]. In summary, even if the application of mature detection technology, the choice of different detection methods may have an impact on the test results. To get one step closer to the clinic, in addition to the existing established detection technologies, on-site detection of miRNAs in the vicinity of the patient is under active research [57]. This will allow us to detect miRNAs more accurately and rapidly in the early stages of disease.

Here, we have briefly reviewed recent studies focusing on miRNAs in obesity-mediated IR. The overall balance of circulating miRNAs is altered in patients with obesity-mediated IR. Compared to that in lean insulin-sensitive patients, the circulation of 65 and 73 miRNAs increased and decreased, respectively, in patients with obesity-mediated IR (374 miRNAs detected in total) [58]. MicroRNA-34a (miR-34a) expression in white adipose tissue of individuals with overweight or obesity is positively correlated with IR and systemic inflammatory parameters [20]. Functional analysis showed that miR-122 is associated with IR, inflammation, and obesity development in individuals with overweight and regulates insulin signaling pathways [59]. Circulating miRNA levels are also influenced by weight loss due to bariatric surgery, exercise, and glucose-reducing therapy [60, 61]. These studies suggest new potential uses of miRNAs as predictive biomarkers for monitoring treatment responses and toward precision medicine for preventing obesity-mediated IR.

In summary, this review focuses on individuals with obesity complicated by IR, focusing on the changes in miRNAs before and after disease onset to predict IR development in individuals with obesity. miRNAs that can potentially become biomarkers from an epigenetic perspective have been summarized.

## MiRNAs: potential biomarkers of obesity-associated IR

**Fig. 2:**
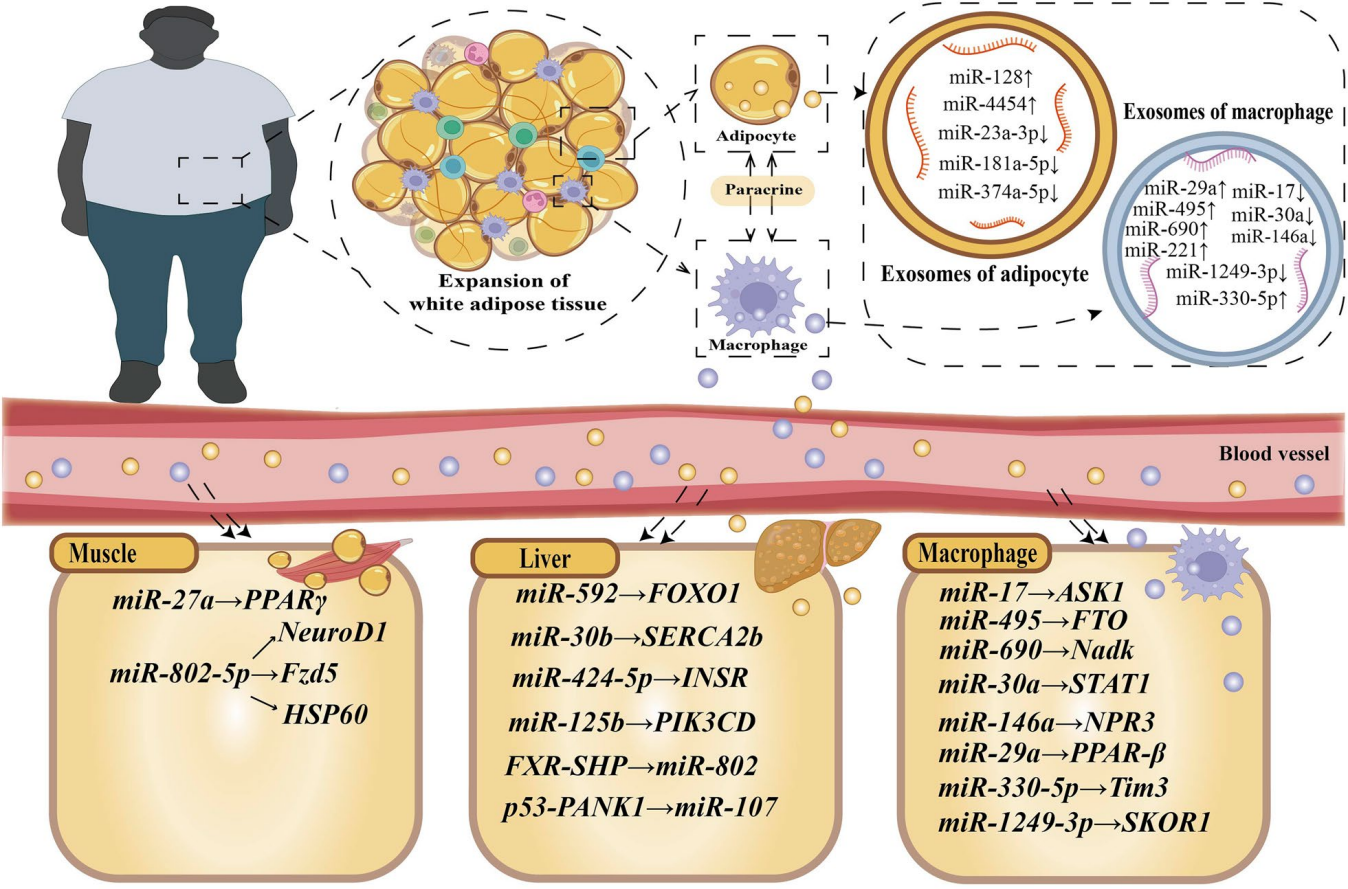
MicroRNAs: biomarkers of obesity-mediated insulin resistance. microRNAs in adipocyte-derived exosomes and macrophage-derived exosomes affect insulin signaling pathways in the liver, skeletal muscle, and macrophages

The process of obesity-mediated IR development is complex and involves multiple signaling pathway mechanisms (Fig. 2). In recent years, many studies have extensively explored the role of miRNAs as biomarkers for predicting IR development in populations with obesity [62–73]. The downstream miRNA targets have also been investigated [74–85]. We summarized miRNAs with biomarker roles according to the study modality and pathogenesis.

### Clinical studies

The clinical studies focusing on the possible relationship between miRNA expression and IR have been summarized according to the study modality (Table 1).

**Table 1:** Clinical research on the changes of microRNA associated with insulin resistance after obesity

Range	Study	Population/age groups	Source	Regulated microRNAs	Role/target
Direct comparative study between MAO and MHO individuals	Fayaz et al. [62]	18 MHO 21 MAO Adults (18–65 years)	Serum	miR-331-3p, miR-452-3p, miR- 485-5p (↑) miR-153-3p, miR-182-5p, miR-4333p(↓)	Biomarkers of HbA1c/IGF1R
	Ghorbani et al. [63]	45 MAO 42 MHO Adults (40–65 years)	Serum	miR-21 (↓)	Biomarkers of obesity-mediated T2D
	Zhang et al. [64]	45 MHO 52 MAO 50 controls Children and adolescents (1–17 years)	Plasma	miR-24-3p (↑)	Biomarkers of metabolic syndrome in children with obesity
	Pan et al. [65]	36 control 36 obese 12 T2D Adults (18–65 years)	Serum	miR-4431 (↑)	Biomarkers of obesity-related glucose metabolism/TRIP10 and PRKD1
	Cerda et al. [66]	6 MAO 6 MHO Adults (18–65 years)	Plasma	miR-155 (↓)	Biomarkers of obesity-related IR and high cardiac metabolic risk/CEBPB
	Lin et al. [67]	7 MHO 8 IR 16 NAFLD Adolescents (10–17 years)	Serum	miR-21-5p, miR-22-3p, miR-150-5p, miR-155-5p, miR-27-3a (↑)	Biomarkers of IR and nonalcoholic fatty liver risk in patients with obesity
Direct comparative study between MAO and healthy individuals	Lin et al. [68]	50 MAO 50 control Adults (40–65 years)	Serum and urine	miR-143-3p (↑)	Biomarkers of obesity-related IR/IGF2R and IGFBP5
	Lin et al. [69]	33 obese Adolescents (10–17 years)	Plasma	miR-30d, miR-221, miR-122 (↑)	Target: Increased levels of miR-30d, miR-221, and miR-122 are associated with obesity-associated IR
	Herrero-Aguayo et al. [70]	Cohort A: 8 male (4 control, 4 obese) Cohort B: 221 male (80 control, 62 obese, 79 overweight) Cohort C: 18 male (10 control, 8 obese) Adults (18–65 years)	Plasma	miR-4454 (↑)	Biomarkers of obesity-related IR and HOMA-index/spliceosome pathway, Akt, AMPK, and ERK
	Arcidiacono et al. [71]	26 MHO 20 control Adults (18–65 years)	Omental visceral adipose tissue, plasma	miR-128 (↑)	Target: Increased miR-128 content correlates with INSR mRNA and protein expression in VAT
	Lozano-Bartolomé et al. [72]	Cohort A:58 obese (28 BMI < 30 kg/m^2^, 30 BMI > 30 kg/m^2^) Cohort B: 101 patients (53 control, 48 obese) Adults (18–65 years)	Adipose tissue, plasma	miR-181a-5p (↓)	Biomarkers of obesity-related insulin resistance/PTEN
	Doumatey et al. [73]	Cohort A:10 MAO, 10 MHO Cohort B:64 MHO, 34 MAO Adults (18–65 years)	Serum	miR-374a-5p (↓)	Target: Decreased miR-374a-5p levels are associated with CCL2 and involved in obesity-associated inflammatory responses

AMPK, Adenosine monophosphate-activated protein kinase; Akt, Protein kinase B; AhR, Aryl hydrocarbon receptor; BMI, Body mass index; CTRL, Control subjects; CCL2, C-C motif ligand 2; CEBPB, CCAAT/enhancer-binding protein beta; ERK, Extracellular regulated protein kinases; HOMA, Homeostasis model assessment; HbA1c, Hemoglobin A1c; IGF2R, Insulin-like growth factor 2 receptor; IGFBP5, Insulin-like growth factor binding protein 5; IGF1R, Insulin-like growth factor 1 receptor; IR, Insulin resistance; INSR, Insulin receptor; miR, microRNA; MHO, Metabolically healthy obese; MAO, Metabolically abnormal obese; NAFLD, Non-alcoholic fatty liver disease; T2D, Type 2 diabetes; PTEN, Phosphatase and tensin homolog; PRKD1, Protein kinase D1; TRIP10, Thyroid hormone receptor interactor 10; VAM, Vesicle associated membrane protein

#### Direct comparative study between MAO and MHO individuals

In recent clinical studies, some investigators have directly compared miRNAs differentially expressed in the peripheral blood of MAO and MHO individuals [62–67]. These studies have substantial implications for mining early biomarkers [62–67].

Several clinical studies have identified differentially expressed miRNAs [62–64]. In a study on 45 MAO and 42 MHO individuals, Ghorbani et al. [63] observed that circulating miR-21 levels were significantly reduced in MAO individuals. In a study on 45 MHO, 52 MAO, and 50 healthy participants, Zhang et al. [64] found elevated miR-24-3p expression in the peripheral serum of MAO individuals. Receiver operating characteristic (ROC) curves showed that miR-24-3p predicted the risk of developing metabolic syndrome in children with obesity (AUC = 0.890) [64]. In addition to screening for individual miRNAs, some studies have conducted extensive screening for miRNAs. In a study on 18 MHO and 21 MAO individuals, Fayaz et al. [62] observed that miR-331-3p, miR-452-3p, and miR-485-5p expression increased and miR-153-3p, miR-182-5p, and miR-433-3p expression decreased in MAO patients than those in MHO patients. However, whether these miRNAs can be combined into a biomarker panel requires further investigation. In a study on 7 MHO, 8 IR, and 16 non-alcoholic fatty liver disease (NAFLD) patients, Lin et al. [67] found that increased serum miR-21-5p, miR-22-3p, miR-150-5p, and miR-155-5p levels could be used to identify NAFLD risk in individuals with obesity. In contrast, elevated serum miR-27-3a levels can be used to determine the IR risk in individuals with obesity [67]. The mode of action of these differentially expressed miRNAs requires further investigation.

Other studies have identified differentially expressed miRNAs and explored the possible mechanisms of miRNA involvement in obesity-related IR [65–67]. In a clinical cohort of 36 healthy individuals, 36 individuals with obesity, and 12 T2D patients, Pan et al. [65]observed a positive correlation between serum miR-4431 levels, body mass index (BMI), and fasting glucose levels. Elevated miR-4431 was mainly distributed in the serum, white adipose tissue, and liver of obese mice [65]. Bioinformatic predictions suggest that miR-4431 impairs glucose metabolism by targeting the thyroid hormone receptor interactor (TRIP) 10/protein kinase (PRK) D1[65]. In a study cohort of 6 MHO and 6 MAO individuals, Cerda et al. [66] observed that miR-155 was differentially expressed in the peripheral blood. Mechanistic studies suggest that miR-155 is involved in IR by targeting CCAAT/enhancer binding protein beta (CEBPB) [66]; Doumatey et al. [73]. Eight differentially expressed miRNAs were screened in the peripheral blood sample of obesity cohort 1 (10 MHO and 10 MAO individuals) and validated in those of obesity cohort 2 (64 MHO and 34 MAO individuals). The miR-374a-5p levels were higher in the MHO individuals than those in MAO individuals [73]. Mechanistic studies suggest that miR-374a-5p may be involved in the inflammatory response by affecting C–C motif ligand (CCL) 2, which further interferes with insulin metabolism in individuals with obesity [73]; visceral adipose tissue (VAT) and subcutaneous adipose tissue (SAT) were collected from patients in a study cohort comprising 28 individuals with BMI ≥ 30 kg/m^2^ and 30 individuals with BMI < 30 kg/m^2^) [72]. Lozano-Bartolomé et al. [72] found that miR-181a-5p expression in the VAT was significantly lower in patients with abnormal glucose tolerance than that in those with standard glucose tolerance. miR-23a-3p expression level in patients with BMI ≥ 30 kg/m^2^ were significantly lower than that in those with BMI < 30 kg/m^2^ [72]. Furthermore, miR-23a-3p and miR-181a-5p were overexpressed. Transient miR-181a-5p and miR-23a-3p overexpression increased pAKT and pAKT substrate 160 kDa (AS160) expression in insulin-stimulated adipocytes by up to 25% [72]. Target studies showed that the insulin signaling gene phosphatase and tamsin homolog (PTEN) and S6K were predicted targets for the joint action of miR-23a-3p and miR-181a-5p [72]. This suggests that miR-181a-5p and miR-23a-3p can synergistically target insulin pathway regulators involved in insulin signaling in adipocytes [72]. In addition, the four serum signatures (HDL, cholesterol, CRP, adiponectin, and miR-181a-5p) could represent a potential biomarker panel that could enable early diagnosis of prediabetic patients [72].

These studies directly compared the differences in miRNAs in peripheral serum between MHO and MAO individuals. Among these, miR-21 [63], miR-331-3p [62], miR-452-3p [62], miR-485-5p [62], miR-153-3p [62], miR-182-5p [62], and miR-433-3p [62] have direct targets of action and can be used as promising diagnostic biomarkers. miR-24-3p [64], miR-155 [66], miR-21-5p [67], miR-22-3p [67], miR-150-5p [67], miR-155-5p [67], miR-27-3a [67], miR-181a-5p [72], and miR-374a-5p [73] can be used as promising prognostic biomarkers. Their predictive validity should be further tested using a large cohort. However, very few studies have focused on such direct comparisons.

#### Direct comparative study between MAO and healthy individuals

In this section, we have discussed the studies comparing differences in peripherally circulated miRNAs between MAO and healthy individuals [68–73]. In a study that included 50 metabolic syndrome patients and 50 healthy participants, Lin et al. [68] found that elevated circulating (serum and urine) miR-143-3p levels were positively associated with IR. The prevalence of metabolic syndrome in participants with high miR-143-3p levels was 6.612 (serum miR-143-3p levels) times and 3.160 (urine miR-143-3p levels) times higher than that in participants with low miR-143-3p levels [68]. Circulating miR-143-3p levels may be downregulated by targeting insulin-like growth factor 2 receptor (IGF2R) and activating the insulin signaling pathway [68]. In a study involving 26 MAO and 20 healthy participants, Arcidiacono et al. [71] found increased miR-128 secretion in the circulation of MAO patients. Mechanistic studies suggest that increased miR-128 inhibits INSR mRNA and protein expression in white adipose tissue, thereby preventing insulin-stimulated glucose uptake by fatty tissue and ultimately inducing systemic IR [71]. Lin et al. [69] found miR-30d, miR-221, and miR-122 to be the most significantly associated with obesity-related IR after preliminarily screening the miRNAs in circulating serum of 33 adolescents with obesity. Among them, miR-221 acts directly on the 3′-untranslated region (UTR) of sirtuin (SIRT) 1 mRNA to promote adipose tissue inflammation and IR by reducing SIRT1 protein levels [86]. The miR-221/SIRT1 pathway may be a potential therapeutic target for reducing adipocyte inflammation during obesity [86]. In a cross-sectional study on 35 children with obesity and 35 healthy children, Ahmadpour et al. [87] found that plasma miR-34a levels were associated with insulin secretion and HOMA-IR. Upregulation of miR-34a stress responsiveness after obesity may inhibit v-SNARE vesicle-associated membrane protein (VAMP) 2 expression in glucose transporter (GLUT) 4 vesicles via the nicotinamide phosphate nucleotidyl transferase (NAMPT)/nicotinic acid phosphate nucleotidyl transferase (NAPRT)/SIRT1/protein tyrosine phosphatase (PTP) 1B axis [87]. In three different cohorts of males with obesity, Herrero-Aguayo et al. [70] found that circulating miR-4454 levels were increased in obesity, associated with key clinical parameters (such as insulin levels and HOMA-IR), and modulated by obesity-controlling interventions (metformin/statin therapy and bariatric surgery). In addition, in vitro data showed that miR-4454 impairs cellular insulin metabolism through key insulin metabolism signaling pathways (protein kinase B (AKT), adenosine-5′-monophosphate-activated protein kinase (AMPK), and extracellular regulatory protein kinase (ERK)) [70].

These studies directly compared the differences in miRNAs in peripheral serum between individuals with obesity and healthy individuals. Among these, miR-34a [87] have direct targets of action and can be used as promising diagnostic biomarkers. miR-143-3p [68] and miR-4454 [70] can be used as promising prognostic biomarkers. The differences in these miRNA expressions should also be validated in a cohort of both MAO and MHO individuals. However, existing studies have low sample sizes and need more direct evidence, and direct comparative studies with large sample sizes are required to screen for more sensitive miRNAs.

### Animal testing

Many experimental studies have focused on the possible relationship between miRNA expression and IR (Table 2), which we summarize according to the mechanism [74–85, 88–96]. As the purpose of animal experiments differs from that of clinical trials, we have categorized the animal experiments according to the different modes of action of miRNAs.

**Table 2:** Animal experiment and cell experiment of insulin resistance-related microRNA changes after obesity

Range	Study	Population	Source	Regulated microRNAs	Target
Obesity–inflammation–IR	Liu et al. [74]	HFD C57BL6 mice NCD lean mice	ATMs-derived exosome Serum	miR-29a (↑)	PPAR-β
	Zhang et al. [75]	Diabetic C57BL6 mice T3-L1 adipocytes cell Mouse macrophage cell	ATMs	miR-17 (↓)	ASK1
	Sun et al. [76]	HFD C57BL/6 mice LFD C57BL/6 mice	ATMs	miR-330-5p (↑)	Tim-3
	Hu et al. [77]	HFD C57BL6 mice NCD C57BL6 mice	ATMs	miR-495 (↑)	FTO
	Ying et al. [78]	HFD C57BL6 mice	M2-polarized bone marrow-derived macrophages secrete miRNA- containing exosomes	miR-690 (↑)	Nadk
	Koh et al. [79]	HFD DIO mice NCD C57BL/6J mice	Subcutaneous white adipose tissue	miR-30a (↓)	STAT1
	Roos et al. [80]	HFD miR-146a−/− mice ND miR-146a−/− mice	White adipose tissue	miR-146a (↓)	NPR3
	Wang et al. [81]	HFD C57BL/6 mice NCD C57BL/6 mice	Natural killer derived exosomes	miR-1249-3p (↓)	SKOR1-SMAD6-TLR4-NF-κB
Obesity–liver–IR	Song et al. [82]	HFD C57BL/6 mice NCD C57BL/6 mice	Liver tissue	miR-592 (↓)	FOXO1, glucose, and lipid metabolism
	Seok et al. [83]	HFD SHP-KO mice NCD FXR-KO mice	Hepatocytes Liver tissue	miR-802 (↑)	FXR-SHP-miR-802
	Yang et al. [84]	HFD C57BL/6 mice NCD C57BL/6 mice	Liver tissue	miR-107 (↑)	P53/PANK1/miR-107
	Price et al. [85]	HFD miR-133−/− mice ND miR-133−/− mice	Plasma Liver tissue	miR-33 (↓)	Lipid metabolism
	Dai et al. [88]	HFD SD rats NCD SD rats	Liver tissue	miR-30b (↑)	SERCA2b, endoplasmic reticulum stress
	Min et al. [89]	HFD C57BL/6N mice NFD C57BL/6N mice PA treated HepG2 cells	Plasma Liver tissue	miR-424-5p (↑)	INSR, insulin signaling and glycogen synthesis
	Du et al. [90]	HFD C57BL/6N mice NFD C57BL/6N mice 10 MAO 10 Control	Liver tissue	miR-125b (↑)	PIK3CD and glucose metabolism
Obesity– Skeletal muscle–IR	Yu et al. [91]	45 children with obesity 45 control children HFD C57BL/6N mice LFD C57BL/6N mice Db/Db mice Db/m mice	Serum Adipose tissue-derived exosomes	miR-27a (↑)	PPAR-γ
	Chen et al. [92]	HFD C57BL/6N mice NFD C57BL/6N mice 3T3-L1 cell	Pancreas Liver White adipose tissue	miR-27a (↑)	PPARγ-PI3K/AKT-GLUT4
Obesity—Islets of langerhans β cell–IR	Zhang et al. [93]	HFD C57BL/6J mice NCD C57BL/6J mice	Islets	miR-802-5p (↑)	NeuroD1 and Fzd5, β cell dysfunction
	Wen et al. [94]	3T3-L1 preadipocytes Cardiac myocytes	Hypertrophic adipocyte-derived exosomes	miR-802-5p (↑)	HSP60
	Xu et al. [95]	HFD C57BL/6J mice NCD C57BL/6J mice	Brown preadipocytes Serum exosomes Islets	miR-26a (↓)	β cell hyperplasia
Obesity–Gut microbiota–IR	Virtue et al. [96]	HFD C57BL/6 mice NCD C57BL/6 mice	Epididymal white adipose tissue Plasma Feces	miR-181 (↑)	Tryptophan derivatives

AKT, Protein kinase B; ATM, Adipose tissue-derived macrophages; ASK, Apoptosis signal-regulated kinase; DIO, Diet-induced obesity; FOXO1, Factor forkhead box O1; Fzd5, Frizzled-5; FXR, Farnesoid X receptor; FTO, Obesity-associated gene; GLUT4, Glucose transporter type 4; HSP60, Heat shock protein 60; HFD, High-fat diet; INSR, Insulin receptor; IRS-1, Insulin receptor substrate 1; IR, Insulin resistance; LFD, Low fat diet; miR-107, MicroRNA-107; MKP5, Mitogen-activated protein kinase phosphatase-5; NPR3, Natriuretic peptide receptor 3; NFD, Normal fat diet; NCD, Normal chow diet; NAFLD, Non-alcoholic fatty liver disease; NFκB, Nuclear factor kappa-B; Nadk, A gene encoding NAD + kinase; PIK3CD, Phosphoinositide 3-kinase catalytic subunit delta; PPAR, Peroxisome proliferator-activated receptor; PI3K, Phosphatidylinositide 3-kinases; PTEN, Phosphatase and tensin homolog; PA, Palmitate; SIRT1, Sirtuin-1; STAT1, Signal transducer and activator of transcription 1; SERCA2b, Sarco/endoplasmic reticulum calcium ATPase 2b; SHP, Small heterodimeric chaperone; SERCA2b, Sarcoplasmic reticulum Ca^2+^-ATPase 2b; SD, Sprague Dawley; SKOR1, SKI family transcriptional corepressor 1; Tim-3, T cell immunoglobulin-3; TLR4, Toll-like receptors 4

#### Obesity–inflammation–IR

Dysregulated M1/M2 macrophage ratio in adipose tissue after obesity induces chronic inflammation, and M1 macrophage activation is associated with IR development [97]. In contrast, M2-type anti-inflammatory macrophages are associated with insulin sensitivity [97]. miR-29a [74], miR-330-5p [76] and miR-495 [77] expression increase in macrophage-derived exosomes in an obese mouse model. MiR-29a regulates cellular insulin sensitivity by targeting peroxisome proliferator-activated receptor (PPAR)-β [74]; miR-330-5p inhibits T cell immunoglobulin (Tim)-3 expression by binding to targets in the Tim-3 3′-UTR [76]. The miR-330-5p/Tim-3 axis may downregulate IR in diabetes patients by enhancing the M2 polarization of macrophages [76], whereas miR-495 promotes macrophage polarization toward the M1 type by targeting the FTO gene in macrophages, thereby participating in IR [77]. In addition to increased expression of miRNAs, miR-17 [75] and miR-690 [78] expression was downregulated in macrophage-derived exosomes. MiR-17 inhibits apoptosis signal-regulated kinase (ASK) 1 expression by targeting its 3′-UTR, blocks macrophage migration, and inhibits pro-inflammatory factor, such as interleukin-6 (IL-6), IL-1β, and tumor necrosis factor-α (TNF-α), secretion [75]. miR-690 may regulate obesity-associated IR by targeting the NAD + kinase-encoding gene (Nadu) [78]. Thus, miR-690 may be a novel epigenetic insulin sensitizer.

In addition to macrophage changes, obesity-associated IR is closely associated with subcutaneous white fat expansion [98]. In a targeted study on SAT, Koh et al. [79] reported low miR-30a levels in the SAT of obese mice. Targeting studies have shown that miR-30a acts directly on the 3′-UTR of signal transducer and activator of transcription 1 (STAT1) [79]. Moreover, miR-30a inhibition significantly induces STAT1 activation and reduces adipocyte sensitivity to interferon (IFN)-γ in obese mice, thereby reducing insulin sensitivity [79]. Notably, the miR-30a expression profile in adipose tissue was independently associated with insulin sensitivity [79]. Roos et al. [80] found that miR-146a gene knockout in mice white adipose tissue reduced insulin sensitivity in adipocytes by targeting the natriuretic peptide receptor 3 (NPR3) gene. Wang et al. [81] found that injecting natural killer (NK) cell-derived exosomes from lean mice into obesity-induced T2D mice improved the systemic IR and inflammatory status of the mice. Mechanistic studies have also shown that miR-1249-3p is overexpressed in NK cell-derived exosomes and attenuates IR and inflammation by regulating the SKI family transcriptional corepressor (SKOR) 1/Smad6/toll-like receptor (TLR) 4/nuclear factor kappa B (NF-κB) axis, providing multiple potential therapeutic targets for T2D [81].

Obesity, chronic inflammation, and IR are strongly linked, and existing studies have revealed the potential of inflammation-related miRNAs in predicting obesity-associated IR [75–81]. The miR-29a- [74], miR-330-5p- [76], miR-495- [77], and miR-30a- [79] associated mechanisms have been investigated thoroughly and can be initially used as candidate early biomarkers for obesity-related IR. However, biomarkers mined based on animal studies need to be further validated in clinical cohorts.

#### Obesity–liver–IR

NAFLD is an important complication of obesity, and the liver is one of the insulin-targeted tissues. Understanding the development of abnormal glucolipid metabolism in the liver of individuals with obesity can help identify new therapeutic targets for metabolic diseases. In an obese mouse model, miR-802 expression increased in the liver [99]. Mechanistic studies suggest that increased miR-802 expression may affect insulin sensitivity and glucose metabolism via the PI3K/AKT pathway, whereas the hepatic energy metabolism pathway (FXR/SHP/miR-802) may provide a novel target for T2D and NAFLD treatment [83]. In contrast, miR-592 reduced the involvement of the hepatic insulin metabolic pathway by acting on forkhead box (FOX) O1, which involves a specific mechanism that remains to be elucidated [82]. In addition, hepatic miR-107 content increases in mice after obesity, accompanied by metabolic reprogramming, and the P53/PTEN-inducible putative kinase (PANK) 1/miR-107 pathway may be an essential link between high-fat diet (HFD)-induced IR and metabolic reprogramming [84]. In a HFD-fed rat model, miR-30b levels were significantly increased in the serum of obese rats with IR than those of obese rats alone [88]. Elevated miR-30b levels may be involved in IR by inhibiting sarcoplasmic reticulum Ca^2+^-ATPase (SERCA) 2b, thereby regulating hepatic endoplasmic reticulum stress, and ultimately IR [88].

MiR-33 is a crucial cholesterol/lipid regulator and one of the targets for atherosclerosis treatment [100]. Price et al. [85] reported that miR-33 knockdown reduced insulin sensitivity in the liver, SAT, and skeletal muscle of HFD-fed mice. Thus, miR-33 may specifically predict IR development in metabolically impaired organisms, and the risk of concomitant metabolic diseases should be monitored when targeting miR-33 for cardiovascular disease treatment [85].

Min et al. [89] found that saturated fatty acids impaired insulin signaling by inhibiting INSR and IRS-1 expression in HepG2 cells. Since miR-424-5p directly targets the INSR gene, miR-424-5p upregulation impairs insulin signaling and insulin-induced glycogen synthesis in hepatocytes by inhibiting INSR [89]. In addition, miR-125b expression was altered in the same cell models [90]. MiR-125b targets phosphatidylinositol-4,5-bisphosphate 3-kinase catalytic subunit delta (PIK3CD), and phosphatidylinositol 3-kinase (PI3K) regulates the insulin regulatory signaling pathway [90]. Thus, miR-125b may be involved in obesity-induced hepatic IR through PI3K regulation [90].

The hepatic metabolic pathway is essential for obesity-related IR. Previous studies have shown that glucose metabolism, lipid metabolism, and energy metabolism in the liver influence obesity-related IR progression, and miRNAs are intimately involved in all three metabolic pathways in the liver [82–85, 88–90]. The mechanisms associated with miR-802 [83], miR-30b [88] and miR-424-5p [89] have been well studied and can be used as preliminary candidates for early diagnostic biomarkers of obesity-related IR.

#### Obesity–skeletal muscle–IR

Insulin targets the skeletal muscles, in addition to the liver, and PPAR-γ deficiency in skeletal muscles is an essential target for IR induction [101]. Yu et al. [91] observed elevated miR-27a expression in the peripheral blood of children and mice with obesity. Mir-27a targets PPAR-γ involved in skeletal muscle insulin signaling [92]. Thus, miR-27a regulates insulin sensitivity in obese mice by targeting the PPARγ-mediated PI3K/Akt signaling pathway [92].

#### Obesity–islet β-cells–IR

The link between obesity and islet β-cell dysfunction is essential for understanding obesity-mediated IR. Zhang et al. [93] detected high miR-802-5p expression in pancreatic islet cells of obese mice, accompanied by reduced insulin levels and impaired glucose tolerance. miR-802-5p knockdown prevented the development of obesity-mediated IR [93]; moreover, miR-802-5p may target the NeuroD1 and frizzled (Fzd) 5 genes involved in insulin regulatory processes [94]. Mechanistic studies have suggested that miR-802-5p present in mast adipocyte-derived exosomes causes cardiac IR by downregulating heat shock protein (HSP) 60 [94].

MiR-26a is reduced in serum exosomes of overweight humans and is inversely correlated with clinical features of T2D [95]. Moreover, miR-26a is down-regulated in serum exosomes and islets of obese mice [95]. Inhibin subunit beta A and DNA methyltransferase 3 alpha are direct targets of miR-26a in the liver[95], where DNA methyltransferase 3 alpha was recently shown to be an epigenetic regulator of IR [102]. In addition to this, previous studies have demonstrated that miR-26a can directly target several regulatory factors critical to liver and fat metabolism that play important roles in obesity and diabetes [103–105]. In addition to the positive mechanism, miR-26a also regulates insulin-induced differentiation of adipose-derived stem cells to adipocytes by modulating the cyclin-dependent kinase (CDK) 5/FOXC2 pathway [106]. These known miR-26a targets, as well as certain undescribed targets, are likely to synergistically regulate the function of miR-26a in IR.

#### Obesity–gut microbiota–IR

Gut microbiota is one of the channels through which the external environment influences biological phenotypes [107]. The gut microbiota maintains metabolic health by regulating white adipose tissue [107]. Virtue et al. [96] reported that in obese mice, environmental alterations decrease tryptophan derivatives secreted by the gut microbiota, further dysregulating miR-181 expression in the host white adipose tissue and inducing obesity-mediated IR and metabolic disturbances. This study suggests that the gut microbiota/miR-181 axis is a target for the clinical treatment of obesity-related metabolic disorders [96]. In addition, oral miR-10a-5p administration alleviates HFD-induced IR by modulating the circadian rhythm of intestinal Lachnospiraceae and its metabolite butyric acid [108].

### Similarities and differences between miRNAs in clinical and preclinical settings

The similarities and differences between miRNAs detected in clinical studies and animal experiments should be discussed. All miRNAs mentioned in this study can currently be detected in human, rat, and mouse tissues or blood without species specificity. The miRNAs detected in clinical studies should be further explored in animal models to explore their specific mechanisms. In terms of variability, metabolic change is a complex process influenced by various social, emotional, and dietary factors. Clinical studies focus on patients, and the observed highly objective changes in miRNAs are the most direct metabolic changes in the body. Thus, these miRNAs have significant potential as biomarkers. In contrast, animal models of obesity are dominated by controlled diets, and there are errors in the observed changes in miRNAs in this context, which require further validation in clinical cohorts.

## Conclusion and outlook

In the last five years, many studies have focused on the role of miRNAs in crosstalk and cueing in metabolic diseases. Moreover, early detection of changes in circulating miRNA profile plays a vital role in identifying the individuals with obesity, who may develop metabolic syndrome [109]. This review summarizes miRNAs identified in clinical trials and animal experiments over the past five years with early cues to obesity-mediated IR. By summarizing the results of clinical trials, we concluded that miR-21 [63], miR-331-3p [62], miR-452-3p [62], miR-485-5p [62], miR-153-3p [62], miR-182-5p [62], miR-433-3p [62], miR-143-3p [68], and miR-4454 [70] have direct targets of action and can be used as promising diagnostic biomarkers. miR-24-3p [64], miR-155 [66], miR-21-5p [67], miR-22-3p [67], miR-150-5p [67], miR-155-5p [67], miR-27-3a [67], miR-181a-5p [72], and miR-374a-5p [73] can be used as promising prognostic biomarkers. We summarized miRNAs from multiple perspectives (including inflammation, liver metabolism, skeletal muscle metabolism, pancreatic β-cells, and gut microbiota) that are early cues for obesity-mediated IR in animal experiments. We focused on miR-29a [74], miR-330-5p [76], miR-495 [77], miR-30a [79], miR-802 [83], and miR-107 [84], which have significant epistatic changes and clear targets. However, the results of animal experiments should serve clinical purposes, the differential expression of these miRNAs should be validated in humans in clinical cohorts. We also describe the targets and possible signaling pathways involved in the actions of miRNAs. Although numerous studies have focused on mechanism mining, most are independent and isolated points that do not constitute a surface. These points should be addressed in the future, and their commonalities should be explored. This will help to further understand the specific mechanisms linking obesity and IR. In addition, the signaling pathway targets could inspire the development of clinical weight loss drugs.

The early predictive validity of single miRNAs as biomarkers is poor, probably because the metabolic pathway between obesity and IR is intricate and involves numerous mechanisms. The miRNAs proposed in this study should be integrated to form a biomarker panel to predict complex metabolic diseases, which might improve the predictability.miRNAs have been detected in the adipose tissue and circulating serum in animal models. Simultaneously, detecting miRNAs in serum samples collected in clinical trials is more convenient. In addition, we wanted to directly compare the differences in miRNA profiles between individuals with MAO and those with MHO to discover more reliable biomarkers. In addition to IR, other obesity-based metabolic complications include chronic inflammation, cardiovascular complications, and osteoporosis. We propose to identify and aggregate early biomarkers for other obesity-associated metabolic complications, thereby accurately predicting the subsequent of obesity-related complications in patients in the early stages of the disease. Detecting early biomarkers will also support clinicians in the early risk stratification of individuals with obesity. In the last five years, studies have focused on children and adolescents. Both genetic and environmental factors influence the expression of these genes in obesity development in children, adolescents, and young adults. Careful analysis of genetic causes, understanding epigenetic changes that influence the obesity epidemic, and obtaining relevant evidence are valuable tools for clinicians dealing with obesity.

## Data Availability

Not applicable.

## Abbreviations

ASK: Apoptosis signal-regulated kinase
AUC: Area under the curve
AS160: AKT substrate of 160 kDa
AKT: Protein kinase B
AMPK: Adenosine 5′-monophosphate-activated protein kinase
BMI: Body mass index
CI: Confidence interval
CCL2: C–C motif ligand 2
CDK5: Cyclin-dependent kinases 5
CEBPB: CCAAT/enhancer-binding protein beta
ERK: Extracellular regulated protein kinases
FOXC2: ForkheadboxC2
Fzd5: Frizzled-5
FOXO1: Forkhead box O1
FXR: Farnesoid X receptor
FFA: Free fatty acids
GLUT4: Glucose transporter 4
HFD: High fat fed
HbA1c: Hemoglobin A1c
HOMA-IR: Homeostasis model assessment for insulin resistance
IRS-1: Insulin receptor substrate 1
IFN-γ: Interferon-gamma
IL: Interleukin
IGF2R: Insulin-like growth factor 2 receptor
INSR: Insulin receptor
IR: Insulin resistance
KLF4: Krtippel like factor 4
MAO: Metabolically abnormal obese
miR-34a: MicroRNA-34a
Mkp5: Mitogen-activated protein kinase phosphatase-5
MHO: Metabolically healthy obese
miRNAs: MicroRNAs
NAMPT: Nicotinamide phosphoribosyltransferase
NAPRT: Nicotinate phosphoribosyltransferase
NAdk: A gene encoding NAD+ kinase
NRP3: Natriuretic peptide receptor 3
NK: Natural killer
NFκB: Nuclear factor kappa-B
NAFLD: Nonalcoholic fatty liver disease
OR: Odds ratio
PANK: PTEN induced putative kinase 1
PI3K: Phosphatidylinositide 3-kinases
PPAR-γ: Peroxisome proliferator-activated receptor gamma
PIK3CD: Phosphatidylinositol-4,5-bisphosphate 3-kinase catalytic subunit delta
PTP1B: Protein tyrosine phosphatase-1B
PPAR-β: Peroxisome proliferator-activated receptor-β
PTEN: Phosphatase and tensin homolog
PRKD1: Protein kinase D1
RT-PCR: Reverse transcription-polymerase chain reaction
ROC: Receiver operating characteristic
SERCA2b: Sarcoplasmic reticulum Ca^2+^-ATPase 2b
STAT1: Signal transducerand activator of transcription 1
SAT: Subcutaneous adipose tissue
SKOR1: SKI family transcriptional corepressor 1
SHP: Small heterodimeric chaperone
SIRT1: Sirtuin 1
TNF-α: Tumor necrosis factor-α
Tim-3: T cell immunoglobulin-3
T2DM: Type 2 diabetes mellitus
TLR4: Toll-like receptors 4
TRIP10: Targeting thyroid hormone receptor interactor 10
UTR: Untranslated region
VAMP2: Vesicle-associated membrane protein 2
VAT: Visceral adipose tissue
